# Maternal Prenatal Urinary Phthalate Metabolite Concentrations and Child Mental, Psychomotor, and Behavioral Development at 3 Years of Age

**DOI:** 10.1289/ehp.1103705

**Published:** 2011-09-06

**Authors:** Robin M. Whyatt, Xinhua Liu, Virginia A. Rauh, Antonia M. Calafat, Allan C. Just, Lori Hoepner, Diurka Diaz, James Quinn, Jennifer Adibi, Frederica P. Perera, Pam Factor-Litvak

**Affiliations:** 1 Department of Environmental Health Sciences, Columbia Center for Children’s Environmental Health,; 2 Department of Biostatistics, and; 3 Department of Population and Family Health, Mailman School of Public Health, Columbia University, New York, New York, USA; 4 National Center for Environmental Health, Centers for Disease Control and Prevention, Atlanta, Georgia, USA; 5 Institute for Social and Economic Research and Policy, Columbia University, New York, New York, USA; 6 Department of Obstetrics, Gynecology and Reproductive Sciences, University of California San Francisco, San Francisco, California, USA; 7 Department of Epidemiology, Mailman School of Public Health, Columbia University, New York, New York, USA

**Keywords:** behavior, child, mental, phthalates, prenatal, psychomotor

## Abstract

Background: Research suggests that prenatal phthalate exposures affect child executive function and behavior.

Objective: We evaluated associations between phthalate metabolite concentrations in maternal prenatal urine and mental, motor, and behavioral development in children at 3 years of age.

Methods: Mono-*n*-butyl phthalate (MnBP), monobenzyl phthalate (MBzP), monoisobutyl phthalate (MiBP), and four di-2-ethylhexyl phthalate metabolites were measured in a spot urine sample collected from 319 women during the third trimester. When children were 3 years of age, the Mental Development Index (MDI) and Psychomotor Development Index (PDI) were measured using the Bayley Scales of Infant Development II, and behavior problems were assessed by maternal report on the Child Behavior Checklist.

Results: Child PDI scores decreased with increasing log*_e_* MnBP [estimated adjusted β-coefficient = –2.81; 95% confidence interval (CI): –4.63, –1.0] and log*_e_* MiBP (β = –2.28; 95% CI: –3.90, –0.67); odds of motor delay increased significantly [per log*_e_* MnBP: estimated adjusted odds ratio (OR) = 1.64; 95% CI: 1.10, 2.44; per log*_e_* MiBP: adjusted OR =1.82; 95% CI: 1.24, 2.66]. In girls, MDI scores decreased with increasing log*_e_* MnBP (β = –2.67; 95% CI: –4.70, –0.65); the child sex difference in odds of mental delay was significant (*p* = 0.037). The ORs for clinically withdrawn behavior were 2.23 (95% CI: 1.27, 3.92) and 1.57 (95% CI: 1.07, 2.31) per log*_e_* unit increase in MnBP and MBzP, respectively; for clinically internalizing behaviors, the OR was 1.43 (95% CI: 1.01, 1.90) per log*_e_* unit increase in MBzP. Significant child sex differences were seen in associations between MnBP and MBzP and behaviors in internalizing domains (*p* < 0.05).

Conclusion: Certain prenatal phthalate exposures may decrease child mental and motor development and increase internalizing behaviors.

Phthalates are a class of high-production-volume chemicals widely used in consumer products (Sathyanarayana 2008). Biomonitoring studies have established unequivocally that exposures in the United States are ubiquitous [Centers for Disease Control and Prevention (CDC) 2011]. Concentrations of certain phthalates in maternal urine during pregnancy have been associated with adverse child cognitive and/or behavioral development (Engel et al. 2010; Miodovnik et al. 2011; Swan et al. 2010). Cross-sectional studies report associations between phthalate metabolites in children’s urine and behavioral problems and reduced IQ postnatally (Cho et al. 2010; Kim et al. 2009). Experimental research is limited but has shown adverse effects of prenatal exposure of rats to di-2-ethylhexyl phthalate (DEHP) and di-*n*-butyl phthalate (DnBP) on pup learning, memory, and behavior (Arcadi et al. 1998; Li et al. 2009; Tanaka 2002, 2005).

Phthalates have short biological half-lives, with most metabolites eliminated within 24 hr (Wittassek and Angerer 2008). Once absorbed, phthalates are rapidly metabolized into monoesters, and some monoesters can undergo further transformations into more hydrophilic oxidative metabolites; metabolites are eliminated mainly in urine (Heudorf et al. 2007). Epidemiologic studies measure phthalate metabolites in urine as internal dosimeters of exposure because urinary enzymatic activity is negligible and most of the metabolites present arise from elimination of endogenous phthalates, rather than from external contamination with phthalates during collection and processing. The aim of the present study was to evaluate the association between child mental, psychomotor, and behavioral development after prenatal exposures to four phthalates: DnBP, diisobutyl phthalate (DiBP), butylbenzyl phthalate (BBzP), and DEHP.

## Materials and Methods

We selected 319 pregnant inner-city women who delivered between 1999 and 2006 from the longitudinal birth cohort of 727 mothers and newborns being conducted by the Columbia Center for Children’s Environmental Health (CCCEH). Enrollment and exclusion criteria have been described previously (Perera et al. 2003). The CCCEH cohort was restricted to nonsmoking women 18–35 years of age who self-identified as either African American or Dominican and who had resided in northern Manhattan or the South Bronx in New York City for at least 1 year before pregnancy. Women were excluded if they used illicit drugs, had diabetes, hypertension, or known HIV, or had their first prenatal visit after the 20th week of pregnancy. The study was approved by the Columbia University Medical Center and CDC institutional review boards (IRBs). Study procedures were explained at enrollment, and each woman signed an IRB-approved consent form. We selected women (*n* = 319) for participation in the present study if phthalate metabolite concentrations had been measured in spot urine samples collected during pregnancy, if the child had completed the Bayley Scales of Infant Development II (BSID-II; *n* = 297) or the mother had completed the Child Behavior Checklist (CBCL; *n* = 286) at the child age 3–year visit, and data were available on model covariates. The 319 subjects did not differ significantly from the remaining subjects in the CCCEH cohort in terms of basic demographics (race/ethnicity, maternal prenatal marital status and education level, household income, proportion on Medicaid or other public assistance) or on child sex, gestational age, and birth weight (all *p*-values > 0.05).

*Questionnaire and medical record data.* A trained bilingual interviewer administered a 45-min questionnaire to each woman in her home during the third trimester of pregnancy, collecting information on demographics, race/ethnicity, home characteristics and residential history, history of active and passive smoking, occupational history, marital status, education and income level, prenatal alcohol and drug use, and maternal psychosocial conditions. We also abstracted information from the mothers’ and infants’ medical records after delivery, including gestational age, infant sex, birth weight, length and head circumference, complications of pregnancy, medication use, and delivery method.

*Urine sample collection and phthalate measurements.* We collected a spot urine sample from the women during the third trimester of pregnancy (mean, 33.1 ± 3.0 weeks of gestation; median, 33 weeks). Samples were stored at Columbia University at –80°C, shipped to the CDC on dry ice, and stored at –70°C until analysis. The urinary phthalate metabolite concentrations were measured at the CDC as previously described (Kato et al. 2005). Each analytical run included calibration standards, reagent blanks, and quality control samples. We used specific gravity to correct for urinary dilution as recommended for phthalates (Hauser et al. 2004). Specific gravity was measured using a handheld refractometer (Atago PAL 10-S; Atago U.S.A. Inc., Bellevue, WA). As a measure of reliability, we calculated intraclass correlation coefficients (ICCs) for the phthalate metabolites in serial spot urine samples collected biweekly from 48 women in the CCCEH cohort over 6–8 weeks late in pregnancy (*n* = 135 samples, 2–4 repeats per woman). Adjusting for specific gravity, ICCs were 0.77 for MBzP, 0.65 for mono-*n*-butyl phthalate (MnBP), and 0.60 for monoisobutyl phthalate (MiBP) and ranged from 0.27 to 0.42 for the DEHP metabolites.

*Measures of child mental, psychomotor, and behavioral development.* The BSID-II (Bayley 1993) provides a developmental quotient (raw score/child chronologic age) from which a continuous Mental Development Index (MDI) and a Psychomotor Development Index (PDI) are generated. The raw scores are converted to a normalized scale with a mean of 100 and standard deviation of 15. Scores can be analyzed continuously (with higher scores indicating better development), or children can be classified as normal or at risk of delay (scores ≤ 85). The child age at the administration of the BSID-II averaged 36.4 ± 1.7 (range, 27–42) months. Cohort children were tested under controlled conditions by trained bilingual research assistants; interrater reliability has been previously described (Rauh et al. 2006). Behavioral problems were measured through maternal report of the 99-item CBCL for children 1.5–5 years of age, which provides an early indicator of potential behavioral problems in young children (Achenbach 2000). The 99 items are summed into seven syndrome scales, with four scales (emotionally reactive, anxious/depressed, somatic complaints, withdrawn) subsequently summed into internalizing behaviors and two (attention and aggressive behaviors) summed into externalizing behaviors. The CBCL scales can be analyzed as continuous scores, or the children can be classified in the normal, borderline, or clinical range based on predetermined cut-points (Achenbach 2000). The child age at the administration of the CBCL averaged 36.6 ± 2.8 (range, 33–48) months.

*Model covariates.* Prenatal psychosocial factors included maternal self-report of hardship during pregnancy (lack of food, clothing, housing, gas or electricity, or medicines) and satisfaction with overall living conditions. Maternal demoralization was measured by the 27-item Psychiatric Epidemiology Research Instrument-Demoralization Scale (Dohrenwend et al. 1978). Maternal intelligence was assessed postnatally by the Test of Non-Verbal Intelligence, third edition (Brown et al. 1990), a 15-min, language-free measure of general intelligence, which is relatively stable and free of cultural bias. The quality of proximal care-taking environment was measured by the Caldwell and Bradley’s (1979) Home Observation for Measurement of the Environment (HOME) scale at child age 38.4 ± 6.2 months. Prenatal alcohol consumption and exposure to environmental tobacco smoke were measured by maternal self-report. Eight polycyclic aromatic hydrocarbons (PAHs) were quantified in 48-hr maternal third-trimester personal air samples and summed (Perera et al. 2003). Bisphenol A (BPA) was measured in the maternal prenatal spot urine samples at CDC as previously described (Ye et al. 2005).

*Statistical analysis.* To examine the relationship between prenatal exposure to the four phthalates (assessed from the urinary metabolite concentrations) and BSID-III and/or CBCL outcomes, we used linear models for the continuous outcomes and logistic models for categorical outcomes. The few phthalate metabolite concentrations below the limit of detection (LOD) were assigned a value of LOD/2. Metabolite concentrations were right skewed and were transformed using the natural logarithm. From a pool of covariates known or suspected of being associated with the phthalate concentrations or BSID-II or CBCL outcomes (Eskenazi et al. 2007; Rauh et al. 2006; Wasserman et al. 2003; Whyatt et al. 2009), we selected those remaining significant or marginally significant (*p* < 0.10) in the regression model for at least one of the outcome variables in the same set. Model covariates for the BSID-II outcomes were child sex (boy vs. girl), race/ethnicity (Dominican vs. African American), the quality of proximal care-taking environment (continuous HOME scale), gestational age (in weeks), maternal marital status (never vs. ever married), maternal prenatal alcohol use (yes/no), and urine specific gravity. Maternal IQ was not controlled because it was not significant once the HOME scale had been added to the model and did not appreciably change the magnitude of the exposure–BSID-II outcome relationships. Covariates in the linear models for CBCL outcomes were child age in months at the time of test administration, child sex, race/ethnicity, maternal IQ (categorized as described below), maternal satisfaction with overall living conditions (yes/no), maternal perceived hardship (yes/no), maternal demoralization (continuous scale), maternal prenatal PAH exposure (categorized as described below), maternal prenatal urinary log*_e_* BPA concentrations, and specific gravity. Multinominal logistic regression was used to analyze the association between the urinary phthalate metabolite concentrations and whether the child fell in the normal, borderline, or clinical range on the CBCL scales. The analyses were conducted only on the four scales that had at least 15 subjects (≥ 5% of the sample) in each cell. The control variables were child’s age in months at test administration, child sex, mother’s satisfaction, maternal demoralization, and urine specific gravity. Urine specific gravity was standardized [(individual subject value – mean value)/SD] before inclusion in all models. Additional variables assessed as confounders but not controlled were maternal education, prenatal environmental tobacco smoke, year and season of urine collection, and umbilical cord lead and chlorpyrifos. To evaluate whether language of test administrations (Spanish vs. English) acted as an effect modifier, we conducted stratified analyses and tested the interaction terms between the phthalate metabolites and language of administration; results were comparable to those presented here, and none of the interaction terms was significant. Missing values for the following five covariates were imputed: *a*) three missing values for maternal demoralization were imputed by a linear regression model with maternal education, maternal satisfaction, and maternal hardship as predictors (model *R*^2^ = 0.15, *n* = 285); *b*) 12 missing observations from the HOME scale were imputed by linear regression with race/ethnicity, maternal education and IQ, and household income as predictors (model *R*^2^ = 0.18, *n* = 294); *c*) five missing observations for maternal hardship were imputed based on a logistic regression with race/ethnicity, maternal demoralization, maternal satisfaction, and prenatal PAH as predictors (model *R*^2^ = 0.18; *n* = 285); *d*) where there were > 5% of missing values (maternal IQ and PAH exposure), we categorized the observed data and added an additional category for missing values. We excluded subjects with missing values for gestational age (*n* = 4) and BPA concentrations (*n* = 9), because these could not be imputed. We also conducted analyses *a*) before imputation of the missing values and *b*) after removing subjects with very dilute urine (specific gravity < 1.007, *n* = 15) or concentrated urine (specific gravity > 1.03, *n* = 7); results were comparable to those presented here. Results from the linear models and the logistic model of BSID-II outcomes are presented for the total cohort and also after stratifying by child sex. Because of small sample sizes in at-risk categories, logistic regression models were adjusted to remove covariates not significantly related to the outcome. Sex differences in the effect of the exposure variable were detected by the Wald test. Sample sizes were too small to stratify by child sex in the multinominal logistic regression analyses for CBCL outcomes. Results were considered significant at *p* < 0.05. Analyses were conducted using SAS (version 9.2; SAS Institute Inc., Cary, NC).

## Results

Table 1 shows subject demographics and distributions of model covariates and outcome variables. Girls scored significantly higher than boys (*p* < 0.01, 2-group test) on both the mental (MDI scores, 93.1 ± 10.9 vs. 88.8 ± 11.5) and psychomotor (PDI scores, 101.4 ± 12.4 vs. 96.3 ± 14.3) scales, whereas boys had significantly higher scores than girls (*p* = 0.03) on attention problems (2.9 ± 2.1 vs. 2.4 ± 1.8). There were no other significant differences between boys and girls on the remaining scales (data not shown). Table 2 shows the distribution of the urinary phthalate metabolite concentrations. Metabolites were detected in 84–100% of the urine samples. As expected, the four DEHP metabolite concentrations, adjusted for specific gravity, were highly correlated (Spearman’s correlation *r*-values ranged from 0.68 to 0.97) and were converted into their molecular weights and summed (ΣDEHP). The correlations between the DEHP and non-DEHP metabolite concentrations were weaker (*r*-values ranged from 0.14 to 0.29). MnBP, MiBP, and monobenzyl phthalate (MBzP) concentrations were also correlated (*r*-values ranged from 0.42 to 0.63), suggesting potential common exposure sources. There was no significant difference in the phthalate metabolites concentration in maternal urine during pregnancy by child sex (data not shown).

**Table 1:** Subject demographics, distribution of model covariates, and outcome variables (*n* = 319).

Characteristic	Value
Maternal age (years) (*n* = 319)		25.5 ± 4.9
Ethnicity (*n* = 319)		
African American		33.5
Dominican or other Hispanic		66.5
Maternal education (*n* = 319)		
< High school degree		37.0
High school diploma or GED		36.7
> High school		26.3
Marital status (*n* = 319)		
Never married		67.4
Married*a*		26.9
Separated, widowed, divorced		5.6
Household income (*n* = 295)		
< $10,000		41.7
$10,000–$30,000		43.0
> $30,000		15.3
Maternal demoralization (*n* = 316)		1.1 ± 0.7
Maternal IQ (*n* = 291)		84.4 ± 13.2
Prenatal PAH in air (ng/m^3^) (*n* = 303)		3.4 ± 9.0
Prenatal BPA in urine (ng/mL) (*n* = 308)		3.2 ± 4.4
Maternal hardship (*n* = 313)*b*		43.1
Maternal satisfaction (% satisfied) (*n* = 319)*c*		27.0
Prenatal alcohol (*n* = 308)*d*		27.9
Child sex (% female) (*n* = 319)		52.7
HOME scale (*n* = 301)		39.3 ± 6.3
Outcome variables		
BSID-II		
MDI (*n* = 297)		91.3 ± 11.3
PDI (*n* = 296)		99.1 ± 13.6
CBCL (*n* = 286)		
Emotionally reactive		1.9 ± 2.1
Anxious/depressed		3.2 ± 2.5
Somatic complaints		2.5 ± 2.4
Withdrawn behavior		2.0 ± 2.2
Sleep problems		2.8 ± 2.4
Attention problems		2.7 ± 1.9
Aggressive problems		10.1 ± 6.9
Internalizing behavior		9.5 ± 6.9
Externalizing behavior		12.8 ± 8.3
Values are mean ± SD or percent. **a**Includes living with same partner for > 7 years. **b**Lack of food, clothing, housing, gas or electricity, or medicines. **c**Maternal self-report of satisfaction with overall living conditions. **d**Reported drinking any alcohol during pregnancy.		

**Table 2:** Distribution of phthalate metabolites (ng/mL) in maternal spot urine during the third trimester of pregnancy (*n* = 319).

Metabolite	Percent > LOD*a*	Geometric mean (95% CI)	Range
DEHP						
Mono-2-ethylhexyl phthalate		84		5.1 (4.3, 6.0)		< LOD to 613
Mono-(2-ethyl-5-hydroxyhexyl) phthalate		100		23.0 (20.1, 26.3)		1.1–1,750
Mono-(2-ethyl-5-oxohexyl) phthalate		100		19.2 (16.8, 22.0)		0.7–1,320
Mono-(2-ethyl-5-carboxypentyl) phthalate		100		40.2 (35.6, 45.4)		3.0–1,840
Non-DEHP						
MBzP		99.7		19.0 (16.4, 22.0)		< LOD to 1,110
MiBP		99.4		9.3 (8.3, 10.5)		< LOD to 374
MnBP		100		38.0 (33.9, 42.6)		0.2–785
**a**LOD was 0.9–1.2 ng/mL for mono-2-ethylhexyl phthalate, 0.3–1.6 ng/ml for mono-(2-ethyl-5-hydoxyhexyl) phthalate, 0.5–1.2 ng/mL for mono-(2-ethyl-5-oxohexyl) phthalate, 0.3–0.6 ng/mL for mono-(2-ethyl-5-carboxypentyl) phthalate, 0.1–1.0 ng/ml for MBzP, 0.3–1.0 ng/mL for MiBP and 0.4–1.1 ng/mL for MnBP.						

No significant associations were found between maternal concentrations of MBzP or ΣDEHP and child mental (MDI) or psychomotor (PDI) scores or mental or motor delay at 3 years of age (all *p*-values > 0.05; Tables 3 and 4). We found a significant inverse association between log*_e_* MnBP [β = –2.81; 95% confidence interval (CI) –4.63, –1.0] and log*_e_* MiBP (β = –2.28; 95% CI: –3.90, –0.67) concentrations and child PDI (Table 3). Among girls, log*_e_* MnBP concentrations were associated with decreases in child MDI (β = –2.67; 95% CI: –4.70, –0.65). The odds of psychomotor delay (scores ≤ 85) increased with concentrations of log*_e_* MnBP [odds ratio (OR) = 1.64; 95% CI: 1.10, 2.44] and log*_e_* MiBP (OR = 1.82; 95% CI: 1.24, 2.66) (Table 4). There were no significant child sex differences in associations between the phthalates and motor delay. There was a significant child sex difference between log*_e_* MnBP and the risk of mental delay (*p* = 0.037; Table 4).

**Table 3:** Estimated coefficient of the predictor (maternal urinary phthalate metabolite concentrations) in the linear models for child MDI and PDI from the BSID‑II.

β-Coefficient (95% CI)			Child sex difference *p*-value
Metabolite (log*e*)	Total	Girls		Boys
PDI		*n* = 296		*n* = 156		*n* = 140		
MnBP		–2.81 ( –4.63, –1.0)**		–2.41 (–4.91, 0.08)		–3.08 (–5.82, –0.33)*		0.72
MiBP		–2.28 (–3.90, –0.67)**		–2.33 (–4.59, – 0.08)*		–2.21 (–4.61, 0.19)		0.94
MBzP		–0.92 (–2.23, 0.40)		–1.05 (–2.77, 0.67)		–0.57 (–2.74, 1.60)		0.73
ΣDEHP		1.31 (–0.26, 2.89)		0.69 (–1.35, 2.73)		2.33 (–0.21, 4.87)		0.32
MDI		*n* = 297		*n* = 157		*n* = 140		
MnBP		–1.12 (–2.62, 0.39)		–2.67 (–4.70, –0.65)**		0.30 (–1.99, 2.59)		0.054
MiBP		–0.28 (–1.62, 1.05)		–1.33 (–3.20, 0.54)		0.59 (–1.40, 2.58)		0.16
MBzP		–0.73 (–1.80, 0.34)		–1.07 (–2.48, 0.33)		–0.45 (–2.23, 1.32)		0.59
ΣDEHP		0.35 (–0.94, 1.64)		–0.22 (–1.90, 1.46)		0.99 (–1.11, 3.09)		0.37
Sex difference was detected by Wald test. Models controlled for specific gravity, race/ethnicity, maternal marital status, maternal prenatal alcohol consumption, gestational age, the quality of proximal care-taking environment (HOME scale), and child sex (total analyses). *p < 0.05, **p ≤ 0.01.								

**Table 4:** OR for child being at risk of mental or psychomotor delay (score ≤ 85) for each log*e* change in phthalate metabolite concentrations in maternal urine.

Metabolite (log*e*)	OR (95% CI)					Sex difference *p*-value
	Total	Girls	Boys			
PDI								
*n* score ≤ 85/total *n *		52/296*a*		19/156*b*		33/140*c*		
MnBP		1.64 (1.10, 2.44)*		1.57 (0.84, 2.94)		1.58 (0.95, 2.61)		0.99
MiBP		1.82 (1.24, 2.66)**		1.98 (1.02, 3.83)*		1.80 (1.13, 2.87)*		0.82
MBzP		1.08 (0.81, 1.44)		1.25 (0.80, 1.95)		0.96 (0.66, 1.39)		0.38
ΣDEHP		0.96 (0.68, 1.36)		1.13 (0.68, 1.87)		0.88 (0.56, 1.40)		0.48
MDI								
*n* score ≤ 85/total *n *		83/297*d*		34/157*e*		49/140*f*		
MnBP		0.93 (0.66, 1.31)		1.44 (0.84, 2.47)		0.68 (0.43, 1.07)		0.037
MiBP		0.89 (0.66, 1.20)		0.98 (0.62, 1.56)		0.87 (0.60, 1.28)		0.71
MBzP		0.89 (0.69, 1.15)		0.94 (0.66, 1.35)		0.89 (0.64, 1.25)		0.83
ΣDEHP		0.79 (0.58, 1.08)		0.95 (0.61, 1.47)		0.71 (0.45, 1.10)		0.35
All logistic regression models controlled for specific gravity. Sex difference was detected by Wald test. **a**Models for PDI ≤ 85 also controlled for maternal marital status, gestational age, quality of proximal care-taking environment (HOME scale), and child sex. **b**Models for PDI ≤ 85 with girls also controlled for HOME scale. **c**Models for PDI ≤ 85 with boys also controlled for HOME scale and maternal marital status. **d**Models for MDI ≤ 85 also controlled for race/ethnicity, maternal marital status, maternal prenatal alcohol consumption, gestational age, HOME scale, and child sex. **e**Models for MDI ≤ 85 with girls also controlled for HOME scale and maternal prenatal alcohol consumption. **f**Models for MDI ≤ 85 with boys also controlled for race/ethnicity and gestational age. *p < 0.05, **p < 0.01.								

No significant associations were found between ΣDEHP metabolite concentrations and any CBCL outcome (all *p*-values > 0.05; Tables 5 and 6). None of the phthalate metabolite concentrations were associated with child sleep problems or with scales in the externalizing domains (all *p*-values > 0.05; data not shown). Among the total cohort, log*_e_* MnBP concentrations were significantly associated with increases in somatic complaints (β = 0.54; 95% CI: 0.19, 0.90), withdrawn behavior (β = 0.40; 95% CI: 0.05, 0.74), and internalizing behaviors (β = 1.45; 95% CI: 0.40, 2.50) (Table 5). Log*_e_* MiBP concentrations were significantly associated with increases in emotionally reactive behavior (β = 0.32; 95% CI: 0.01, 0.62). Log*_e_* MBzP concentrations were associated with significant increases in withdrawn behavior (β = 0.31; 95% CI: 0.07, 0.55) and internalizing behaviors (β = 0.83; 95% CI: 0.11, 1.56). Associations between phthalate concentrations and internalizing behaviors varied somewhat by child sex. Among boys only, log*_e_* MnBP concentrations were significantly associated with emotionally reactive behavior, somatic complaints, withdrawn behavior, and internalizing behaviors (Table 5). Among girls only, log*_e_* MBzP concentrations were significantly associated with anxious/depressed behavior, somatic complaints, withdrawn behavior, and internalizing behaviors. The child sex difference was significant for MnBP on emotionally reactive behavior (*p* = 0.03) and for MBzP on anxious depressed behavior (*p* = 0.035), somatic complaints (*p* = 0.01), and internalizing behaviors (*p* = 0.04), but not for the other scales (*p*-values ranged from 0.12 to 0.99; Table 5).

**Table 5:** Estimated coefficient of the predictor (maternal urine phthalate concentrations) in the linear models for internalizing behaviors on the CBCL when children were 3 years of age.

Behavior/metabolite (log*e*)	β-Coefficient (95% CI)					Sex difference *p*-value
	Total cohort (*n* = 277)	Girls (*n* = 148)	Boys (*n* = 129)			
Emotionally reactive								
MnBP		0.25 (–0.09, 0.58)		–0.02 (–0.50, 0.45)		0.71 (0.22, 1.19)**		0.03
MiBP		0.32 (0.01, 0.62)*		0.34 (–0.11, 0.78)		0.42 (–0.005, 0.85)		0.79
MBzP		0.21 (–0.02, 0.44)		0.26 (–0.05, 0.57)		0.34 (–0.008, 0.69)		0.72
ΣDEHP		–0.0002 (–0.27, 0.27)		0.07 (–0.30, 0.44)		–0.007 (–0.5, 0.4)		0.79
Anxious/depressed								
MnBP		0.26 (–0.11, 0.65)		0.41 (–0.11, 0.94)		0.17 (–0.40, 0.75)		0.54
MiBP		0.12 (–0.23, 0.47)		0.16 (–0.34, 0.66)		0.12 (–0.38, 0.61)		0.91
MBzP		0.22 (–0.04, 0.48)		0.51 (0.17, 0.85)**		–0.05 (–0.46, 0.35)		0.035
ΣDEHP		0.18 (–0.13, 0.49)		0.18 (–0.23, 0.59)		0.19 (–0.32, 0.70)		0.98
Somatic complaints								
MnBP		0.54 (0.19, 0.90)**		0.43 (–0.06, 0.91)		0.77 (0.21, 1.33)**		0.35
MiBP		0.25 (–0.08, 0.58)		0.24 (–0.22, 0.70)		0.31 (–0.18, 0.81)		0.83
MBzP		0.09 (–0.15, 0.34)		0.42 (0.10, 0.73)**		–0.23 (–0.63, 0.17)		0.01
ΣDEHP		–0.06 (–0.35, 0.23)		–0.08 (–0.45, 0.30)		–0.12 (–0.62, 0.39)		0.90
Withdrawn behavior								
MnBP		0.40 (0.05, 0.74)*		0.47 (–0.03, 0.98)		0.56 (0.09, 1.03)*		0.79
MiBP		0.28 (–0.04, 0.60)		0.47 (–0.007, 0.94)		0.36 (–0.05, 0.77)		0.74
MBzP		0.31 (0.07, 0.55)**		0.61 (0.29, 0.93)^#^		0.24 (–0.09, 0.58)		0.12
ΣDEHP		–0.04 (–0.32, 0.25)		–0.04 (–0.44, 0.36)		0.15 (–0.28, 0.57)		0.52
Internalizing behavior								
MnBP		1.45 (0.40, 2.50)**		1.29 (–0.15, 2.72)		2.21 (0.66, 3.76)**		0.39
MiBP		0.97 (–0.002, 1.94)		1.20 (–0.15, 2.55)		1.21 (–0.16, 2.56)		0.99
MBzP		0.83 (0.11, 1.56)*		1.79 (0.88, 2.69)**		0.29 ( –0.83, 1.42)		0.04
ΣDEHP		0.08 (–0.78, 0.95)		0.13 (–0.99, 1.26)		0.21 (–1.21, 1.63)		0.93
Sex difference was detected by Wald test. Models controlled for ethnicity, maternal IQ, maternal demoralization, maternal hardship and maternal satisfaction during pregnancy, maternal prenatal exposure to PAH and BPA, child sex, child age in months at the time of the CBCL administration, and specific gravity. *p < 0.05, **p ≤ 0.01, ^#^*p* < 0.001.								

**Table 6:** OR (95% CI) for child scoring in the borderline or clinical compared with normal range on internalizing behaviors for each log*e* unit increase in maternal phthalate metabolite concentrations (*n* = 286).

Behavior/metabolite (log*e*)	Borderline	Clinical
Somatic complaints		*n* = 35		*n* = 18
MnBP		1.32 (0.84, 2.08)		1.37 (0.73, 2.56)
MiBP		1.29 (0.84, 1.99)		0.76 (0.42, 1.36)
MBzP		0.83 (0.59, 1.15)		1.20 (0.78, 1.86)
ΣDEHP		0.88 (0.58, 1.33)		0.96 (0.57, 1.61)
Withdrawn behavior		*n* = 15		*n* = 23
MnBP		0.60 (0.31, 1.16)		2.23 (1.27, 3.92)**
MiBP		0.81 (0.44, 1.51)		1.62 (0.97, 2.73)
MBzP		0.79 (0.48, 1.28)		1.57 (1.07, 2.31)*
ΣDEHP		0.67 (0.35, 1.30)		0.98 (0.62, 1.55)
Internalizing behavior		*n* = 31		*n* = 37
MnBP		1.31 (0.82, 2.10)		1.44 (0.92, 2.25)
MiBP		1.98 (1.24, 3.23)**		1.41 (0.91, 2.18)
MBzP		1.38 (1.01, 1.90)*		1.43 (1.01, 1.90)*
ΣDEHP		1.26 (0.85, 1.86)		1.19 (0.82, 1.72)
Models controlled for maternal demoralization and maternal satisfaction during pregnancy, child sex, child age in months at the time of the CBCL administration, and specific gravity. *p < 0.05, **p ≤ 0.01.				

Table 6 shows estimated ORs for exposure and scores on the borderline and clinical ranges on the CBCL. We observed significantly increased ORs for the association between MnBP and MBzP concentrations and scores in the clinical range for withdrawn behavior (for each log unit increase, respectively: OR = 2.23; 95% CI: 1.27, 3.92; and OR = 1.57; 95% CI: 1.07, 2.31). Finally, we found increased ORs per log unit increase for scoring in the borderline range for internalizing behaviors related to MiBP concentrations (OR = 1.98; 95% CI: 1.24, 3.23) and MBzP concentrations (OR = 1.38; 95% CI: 1.01, 1.90) and for scoring in the clinical range on internalizing behaviors related to MBzP concentrations (OR = 1.43; 95% CI: 1.01, 1.90).

## Discussion

This is one of the few epidemiologic studies to estimate effects of prenatal phthalate exposures on child cognitive and behavioral development and, to our knowledge, is the only study to look at these associations during the preschool years. MnBP and MiBP metabolite concentrations during pregnancy were significantly associated with decreases in psychomotor development and with increased odds of psychomotor delay. In girls, but not boys, maternal prenatal MnBP was also associated with a significant decrease in the child’s mental development at 3 years of age. Further, MnBP, MiBP, and MBzP were significantly associated with increases in a number of behavioral problems in the internalizing domains and increased odds that the child would score in the clinical range. Geometric mean concentrations of the phthalate metabolites measured here were 1.3–2.7 times higher than in a representative sample of pregnant U.S. women sampled in 2003–2004 (Woodruff et al. 2011).

In addition to our study, Engel et al. (2010) evaluated associations between prenatal phthalate metabolite concentrations and outcomes on the Behavioral Rating Inventory of Executive Function and the Behavior Assessment System for Children-Parent Rating Scales (BASC-PRS) when children were 4–9 years of age (*n* = 171). The metabolites were divided into high-molecular-weight phthalates (HMWPs; predominantly DEHP metabolites) and low-molecular-weight phthalates (LMWPs; including MnBP, MiBP). No significant associations were found between urinary concentrations of the HMWP metabolites and most of the outcomes, except for adaptability. However, urinary concentrations of LMWPs were significantly associated with poorer scores on aggression, attention, conduct problems, depression, and externalizing problems. Most of these associations were specific to boys. LMWP concentrations were also positively associated with poorer scores on the global executive composite index and emotional control scale, as well as social cognition, social communication, and social awareness (Miodovnik et al. 2011). A second longitudinal birth cohort study by Swan et al. (2010) evaluated associations between maternal prenatal phthalate metabolite concentrations and behavior in children 3.6–6 years of age (*n* = 145) on the Pre-School Activities Inventory, a validated instrument to assess sexually dimorphic play behavior. MnBP, MiBP, and the DEHP metabolite concentrations were associated with less masculine play behavior among boys. Two cross-sectional studies reported that DEHP and/or DnBP metabolites in child urine were positively associated with behavioral problems and inversely associated with IQ among Korean children (Cho et al. 2010; Kim et al. 2009). Comparisons of these results to our findings are challenging because study designs, metabolite concentrations, and outcome measures differed and the children were evaluated at different ages. The latter could be particularly important because parents’ awareness of the internal states of their children may vary between school-age and younger children. However, significant correlations between anxious/depressed, somatic complaints, and withdrawn and internalizing behaviors by parental report on the CBCL and depression and attention problems by parental report on the BASC-PRS have been seen among both preschoolers and older children (Doyle et al. 1997; Reynolds and Kamphaur 1992), providing evidence of some comparability between our findings and those of Engel et al. (2010) discussed above.

Experimental studies in laboratory rodents are similarly limited but have found significant associations between prenatal DEHP exposure and significant increases in time to perform the beam walking test (Arcadi et al. 1998), decrease in surface righting, and acceleration in the swimming direction tests (Tanaka 2002, 2005); and between prenatal DnBP exposure and depressed surface righting, shortened forepaw grip time, and inhibition in spatial learning and memory at low doses, but enhanced spatial learning and memory at high doses. Sex × treatment effects were seen for a number of domains, and males appeared to be more sensitive (Li et al. 2009).

In terms of potential mechanism, associations between exposures to DEHP, DnBP, and BBzP and modulation of thyroid function or reductions in circulating thyroid hormone levels have been seen in experimental studies (Breous et al. 2005; Hinton et al. 1986; Howarth et al. 2001; O’Connor et al. 2002; Pereira et al. 2007; Poon et al. 1997; Price et al. 1998; Sugiyama et al. 2005), as well as several epidemiologic studies (Boas et al. 2010; Huang et al. 2007; Meeker and Ferguson 2011; Meeker et al. 2007), and could be one mechanism given the critical role that thyroid hormone plays in fetal and early postnatal brain development (Attree et al. 1992; Hendrich et al. 1984; Porterfield and Hendrich 1993). Furthermore, modulation of testosterone production by phthalates in the male fetus could be another potential mechanism whereby the compounds could disrupt sexually dimorphic behaviors. Experimental data have established that DnBP, DiBP, BBzP, and DEHP are all equally potent at inhibiting testosterone production in male rats during fetal development (Howdeshell et al. 2008) and that this disruption results in abnormalities in genital development (Swan 2008). However, testosterone also plays a critical role in male brain development. Testosterone synthesized by the testis diffuses into the brain, where it is converted to estradiol by aromatase in specific male brain regions (Roselli et al. 2009). The estradiol is thought to organize the brain along a masculine phenotype, with resultant male sexual behaviors (Wu et al. 2009). Reduction of testosterone production by phthalates during fetal development has been hypothesized as a mechanism for the feminization in play behavior observed among boys (Swan et al. 2010). Preliminary data also suggest that phthalates can modulate aromatase activity itself, and this could be yet a third potential mechanism affecting brain development. Estradiol is synthesized de novo in both male and female brains from cholesterol, a reaction catalyzed by aromatase, and is essential for brain development in both sexes (Bakker and Brock 2010; McCarthy 2009; Roselli et al. 2009). Only one prior experimental study has shown a link between phthalate exposure and modulations of aromatase activity in the developing brain (Andrade et al. 2006). Phthalates have also been shown to modulate aromatase activity or expression in other tissues (Adibi et al. 2010; Lovekamp-Swan and Davis 2003). Finally, experimental studies suggest that prenatal DEHP exposure alters transfer of essential fatty acids across the placenta and decreases lipid content in fetal brains. This could be another mechanism whereby phthalate exposure might alter brain development (Xu et al. 2007, 2008).

In addition to the uncertainty over potential mechanisms whereby prenatal phthalate exposure might be affecting child mental, motor, or behavior development, additional limitations in our study results should be noted. In particular, whereas prior results, as well as our own study findings, suggest that phthalate–outcome relationships are likely to be sexually dimorphic, mechanistic understanding of the child sex × phthalate interactions is in its infancy. It is certainly possible that mothers are more likely to be concerned about, and thus report, internalizing behaviors in boys compared with girls. However, reporting bias is unlikely to have influenced our results because MnBP was associated with internalizing behaviors in boys but not in girls, whereas MBzP was associated with internalizing behaviors in girls but not boys, and a number of the child sex differences were statistically significant. More important, assessment of mental, motor, and behavioral development during the preschool years is challenging (Burchinal et al. 2000; Sternberg et al. 2001). Our findings may also be compromised by the reliability of the biomarkers used to characterize DEHP exposure, because the ICCs in repeat urine samples were lower for the DEHP metabolites than for metabolites of the other three phthalates examined. Moreover, prior epidemiologic studies are extremely limited, and none has estimated effects of prenatal phthalate exposure on these outcomes during the preschool years, making the comparison of our findings to the prior results difficult.

## Conclusion

Results presented here suggest that prenatal exposure to DnBP, DiBP, and BBzP may adversely affect child mental, motor, and behavioral development during the preschool years. These findings raise a public health concern but, given the limitations discussed above, should be interpreted with caution, and additional research is warranted. This is especially true in light of recent epidemiologic findings among elementary school-age children showing significant negative correlations between internalizing behaviors (anxious/depressed and withdrawn symptoms) on the CBCL and intellectual function, language, visual construction skills, attention, processing speed, executive function, aspects of learning and memory, psychomotor coordination, and basic academic skills (Lundy et al. 2010). We will continue to follow children in the present cohort to assess associations between prenatal as well as postnatal phthalate exposures and child mental, motor, and behavioral development during the elementary school years.
